# Identification of a protein signature for predicting overall survival of hepatocellular carcinoma: a study based on data mining

**DOI:** 10.1186/s12885-020-07229-x

**Published:** 2020-08-03

**Authors:** Zeng-hong Wu, Dong-liang Yang

**Affiliations:** Department of Infectious Diseases, Union Hospital, Tongji Medical College, Huazhong University of Science and Technology, Wuhan, 430022 China

**Keywords:** Hepatocellular carcinoma, Proteomics, CPTAC, TCPA, TCGA, Prognosis

## Abstract

**Background:**

Hepatocellular carcinoma (HCC), is the fifth most common cancer in the world and the second most common cause of cancer-related deaths. Over 500,000 new HCC cases are diagnosed each year. Combining advanced genomic analysis with proteomic characterization not only has great potential in the discovery of useful biomarkers but also drives the development of new diagnostic methods.

**Methods:**

This study obtained proteomic data from Clinical Proteomic Tumor Analysis Consortium (CPTAC) and validated in The Cancer Proteome Atlas (TCPA) and TCGA dataset to identify HCC biomarkers and the dysfunctional of proteogenomics.

**Results:**

The CPTAC database contained data for 159 patients diagnosed with Hepatitis-B related HCC and 422 differentially expressed proteins (112 upregulated and 310 downregulated proteins). Restricting our analysis to the intersection in survival-related proteins between CPTAC and TCPA database revealed four coverage survival-related proteins including *PCNA, MSH6, CDK1,* and *ASNS*.

**Conclusion:**

This study established a novel protein signature for HCC prognosis prediction using data retrieved from online databases. However, the signatures need to be verified using independent cohorts and functional experiments.

## Background

Hepatocellular carcinoma (HCC), is the fifth most common cancer in the world and the second most common cause of cancer-related deaths. Over 500,000 new HCC cases are diagnosed each year [1]. Viral hepatitis and nonalcoholic steatohepatitis are the most common causes of cirrhosis which underlies approximately 80% of cases of HCC [2]. HCC prognosis remains a challenge due to the recurrence of HCC and the 5-year overall survival rate is only 34 to 50% [3]. Despite the rapid advancements in medical technology, there are still no effective treatment strategies for HCC patients [4]. Byeno et al [5] reported that based on long-term survival data, the serum OPN and DKK1 levels in patients with liver cancer can be used as novel biomarkers that predict prognosis. Other serum markers, such as alpha-fetoprotein (AFP) and alkaline phosphatase (ALP or AKP), have also been reported in clinical practice, however, these markers lack sufficient sensitivity and specificity [6]. Therefore, it is necessary to find effective biomarkers essential for diagnosis and treatment for HCC.

Proteomics is a field of research that studies the proteins at a large-scale level. Biomarker analysis uses high-throughput sequencing technologies in proteomics and genomics. Mass spectrometry-based targeted proteomics has been used to set up multiple omics. Mass spectrometry-based identification of matching or homologous peptide identification can further refine gene model [7]. This allows for an in-depth analysis of host-pathogen interactions. Combining advanced genomic analysis with proteomic characterization not only has great potential in the discovery of useful biomarkers but also drives the development of new diagnostic methods and therapies. Proteogenomic studies have enabled the exploration of the prognosis of cancer progression, however, its role and mechanism remain unclear. Chiou et al [8] used integrated proteomic, genomic, and transcriptomic techniques to obtain protein expression profiles from HCC patients. This study found that S100A9 and granulin protein markers were associated with tumorigenesis and cancer metastasis in HCC. Similarly, Chen et al [9] using a proteomic approach found that curcumin/β-cyclodextrin polymer (CUR/CDP) inclusion complex exhibited inhibitory effects on HepG2 cell growth. Over the last few years, integrative tools useful in executing complete proteogenomics analyses have been developed. In this study, we systematically evaluated the prognostic protein signature for the prediction of overall survival (OS) for HCC patients. The availability of high-throughput expression data has made it possible to use global gene expression information to analyze the genetic and clinical aspects of HCC patients. Therefore, in this study, protein data from Clinical Proteomic Tumor Analysis Consortium (CPTAC) and validated in The Cancer Proteome Atlas (TCPA) and the cancer genomic maps (TCGA) dataset was used to identify HCC biomarkers and the dysfunctional of proteogenomics.

## Methods

### Data collection

CPTAC is a public repository of well-characterized, mass spectrometry (MS)-based and targeted proteomic assays, useful in characterizing the protein inventory in tumors by leveraging the latest advances in mass spectrometry-based discovery proteomics [10]. TCPA is a user-friendly data portal that contains 8167 tumor samples in total, which consists primarily of TCGA tumor tissue samples and provides a unique opportunity to validate the TCGA data and identify model cell lines for functional investigations [11]. TCGA has generated multi-platform cancer genomic data and generated some proteomic data using the Reverse Phase Protein Array (RPPA) platform, measuring protein levels in tumors for about 150 proteins and 50 phosphoproteins [12]. In this study, proteomics data was downloaded from TCPA (level 4) and combined with clinical data from TCGA, and comprehensive analysis of proteomics performed through CPTAC.

### Establishing the prognostic gene signature

Univariate Cox regression analysis was performed to identify prognostic genes and establish their genetic characteristics. The prognostic gene signature was demonstrated as risk score = (CoefficientmRNA1 × expression of mRNA1) + (CoefficientmRNA2 × expression of mRNA2) + ⋯ + (CoefficientmRNAn × expression mRNAn). Based on the median risk score, the patients were classified into the low-risk (<median) group and a high-risk (≥median) group. The Kaplan–Meier survival analysis was used to analyze the survival difference between the high and low groups.

### Building and validating a predictive nomogram

Nomograms are often used to predict the prognosis of cancer. Mainly because they can simplify statistical prediction models to a single numerical assessment of the probability of an event (such as relapse or death) depending on the condition of an individual patient [13]. A receiver operating characteristic (ROC) curve was plotted over time to assess the prediction accuracy of prognostic signals in HCC patients. Univariate and multifactorial Cox regression analysis was used to analyze the relationship between gene clinicopathological parameters.

### Statistical analysis

Statistical analyses were performed using R (version 3.5.3) and R Bioconductor software packages. Benjamini–Hochberg’s method was used to convert *P* values to FDR. Perl language was used for data matrix and data processing and a *P* value less than 0.05 was used. The identification of differentially expressed proteins between HCC and non-cancerous samples in CPTAC used |log2FC| > 1 and a *P-*value < 0.05 was considered to be statistically significant.

## Results

### Establishment of the prognostic gene signatures

**Fig. 1:**
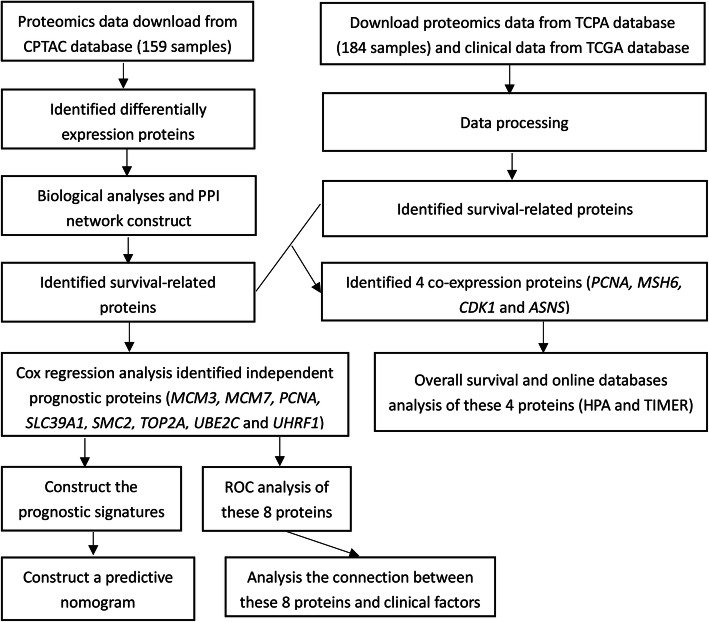
The flow chart showing the scheme of the study on protein prognostic signatures

**Fig. 2:**
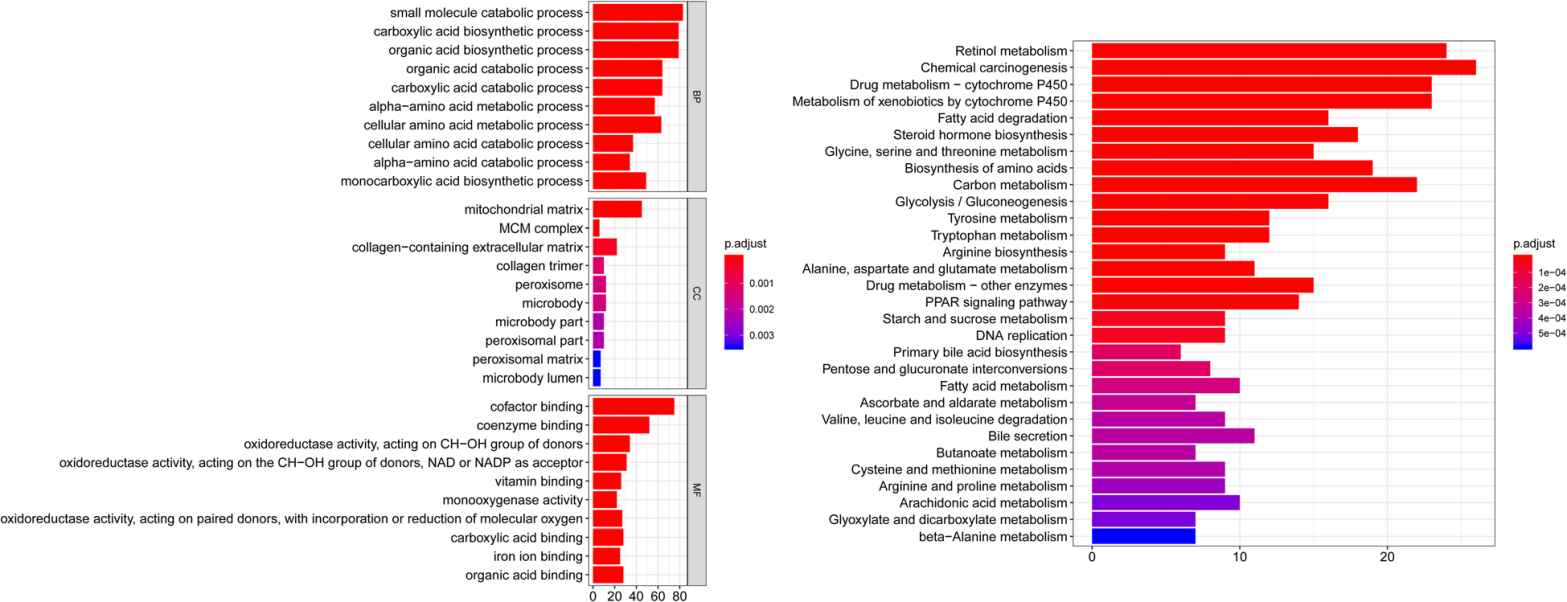
Functions of the identified differentially expressed proteins using GO enrichment and KEGG pathway analysis

Figure 1 presents a flow chart of this study scheme. A total of 159 patients diagnosed with Hepatitis-B related HCC [14] (159 tumor tissues and 159 paratumor tissues Table S1) and 422 differentially proteins (112 upregulated and 310 downregulated Table S2) were identified from the CPTAC database. To analyze the function of the identified differentially expressed proteins, biological analyses were performed using gene ontology (GO) enrichment and KEGG pathway analysis. GO analysis revealed that the GO terms related to biological processes (BP) of differentially expressed proteins were enriched in fatty acid biosynthesis and catabolism, molecular function (MF) were mainly enriched in cofactor binding, coenzyme binding, vitamin binding, monooxygenase activity, carboxylic acid-binding, iron ion binding, and organic acid binding and cell component (CC) were mainly enriched in the mitochondrial matrix, MCM complex, collagen trimer, peroxisome, microbody, microbody part, peroxisomal part, peroxisomal matrix, and microbody lumen. KEGG pathway analysis revealed that the differentially expressed proteins were mainly enriched in retinol metabolism, chemical carcinogenesis, drug metabolism-cytochrome P450, fatty acid degradation, arginine biosynthesis, PPAR signaling pathway and other metabolic pathways (Fig. 2).

### Protein-protein interaction (PPI) network construction and module analysis

To further explore the relationship between differentially expressed proteins at the protein level, the PPI network was constructed based on the interactions of differentially expressed proteins. A total of 542 interactions and 236 nodes were screened to establish the PPI network and the top five most contiguous nodes between genes were *CDK1, AOX1, CYP2E1, CYP3A4,* and *TOP2A* (Table S3-S4).

### Survival analysis

**Fig. 3:**
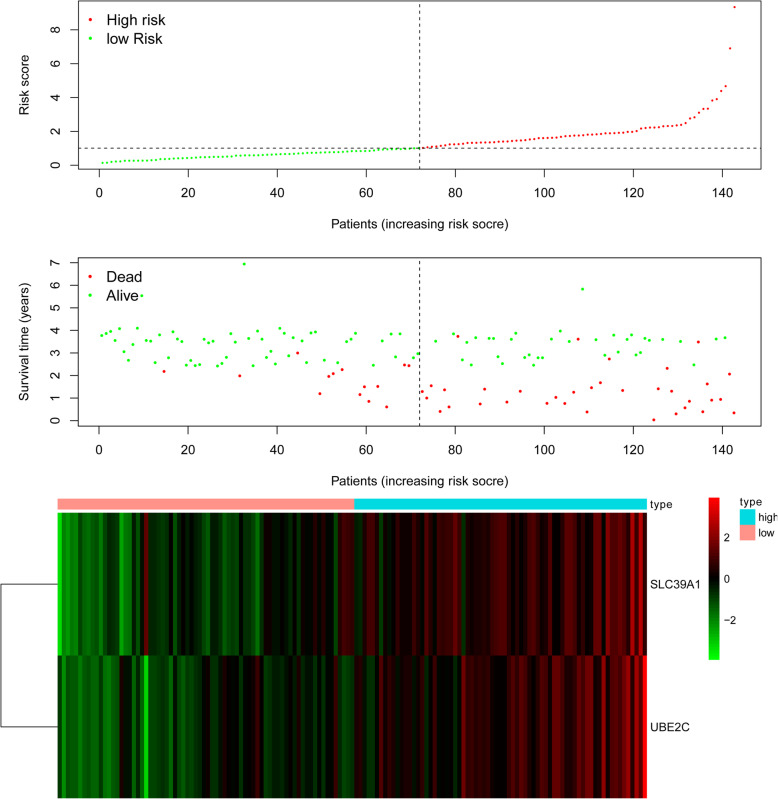
Detailed prognostic signature information of HCC groups

Survival data was extracted from HCC patients in CPTAC and used to perform univariate Cox regression analysis. The expression of survival-related proteins revealed 105 survival-related proteins (*P*<0.05, Table S5). Univariate and multivariate Cox regression analysis was performed on the clinical factors and survival-related proteins and 41 proteins that can act as independent prognostic factors for OS were identified (Table S6-S7). ROC curves were used to investigate the use of the protein patterns as early predictors of HCC incidence. This model demonstrated that 8 proteins (*MCM3, MCM7, PCNA, SLC39A1, SMC2, TOP2A, UBE2C,* and *UHRF1*) had an AUC value above 0.7 (Table S8). Table S9 presents detailed information about the relationship between the 8 proteins and clinical factors. The 8 proteins were used to build a prognostic model, and the median risk score set as the threshold to divide the cohort into high-risk and low-risk groups. The detailed prognostic signature information of the HCC group is shown in Fig. 3**.**

### Building a predictive nomogram

**Fig. 4:**
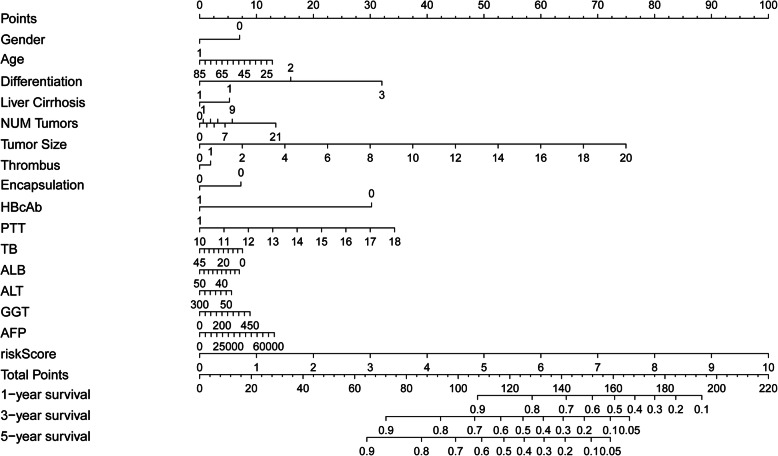
Nomogram constructed using clinical pathology data and prognosis model

A Nomogram was constructed by involving clinical pathology and prognosis models. The LASSO logistic regression algorithm was used to select the most important prediction markers which greatly contributed to the final prediction model. The model included features in CPTAC: gender, age, tumor differentiation, history of liver cirrhosis, number of tumors, tumor size, tumor thrombus, tumor encapsulation, HBcAb, AFP, PTT, TB, ALB, ALT, and GGT (Fig. 4**)**. The use of the prognostic model and clinical pathology data can improve the sensitivity and specificity of 1-, 3-, and 5-year OS prediction.

### Immunohistochemistry analysis

**Fig. 5:**
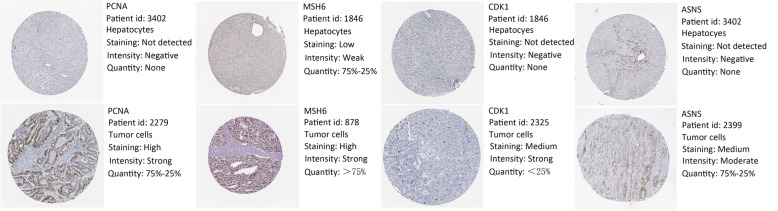
Representative protein expressions of PCNA, MSH6, CDK1, and ASNS explored in the HPA database

**Fig. 6:**
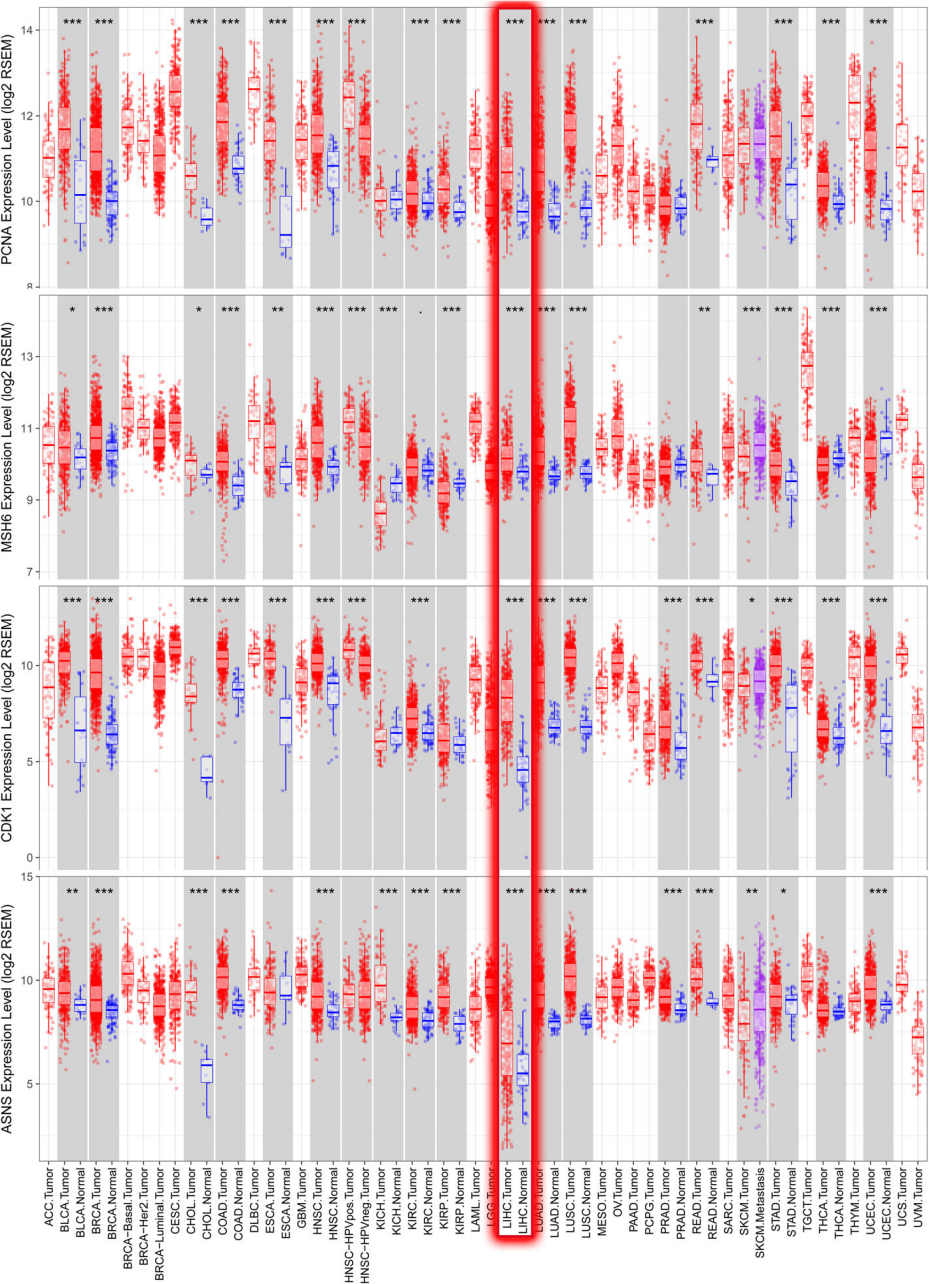
PCNA, MSH6, CDK1, and ASNS proteins significantly overexpressed in HCC. LIHC: Liver Hepatocellular Carcinoma

**Fig. 7:**
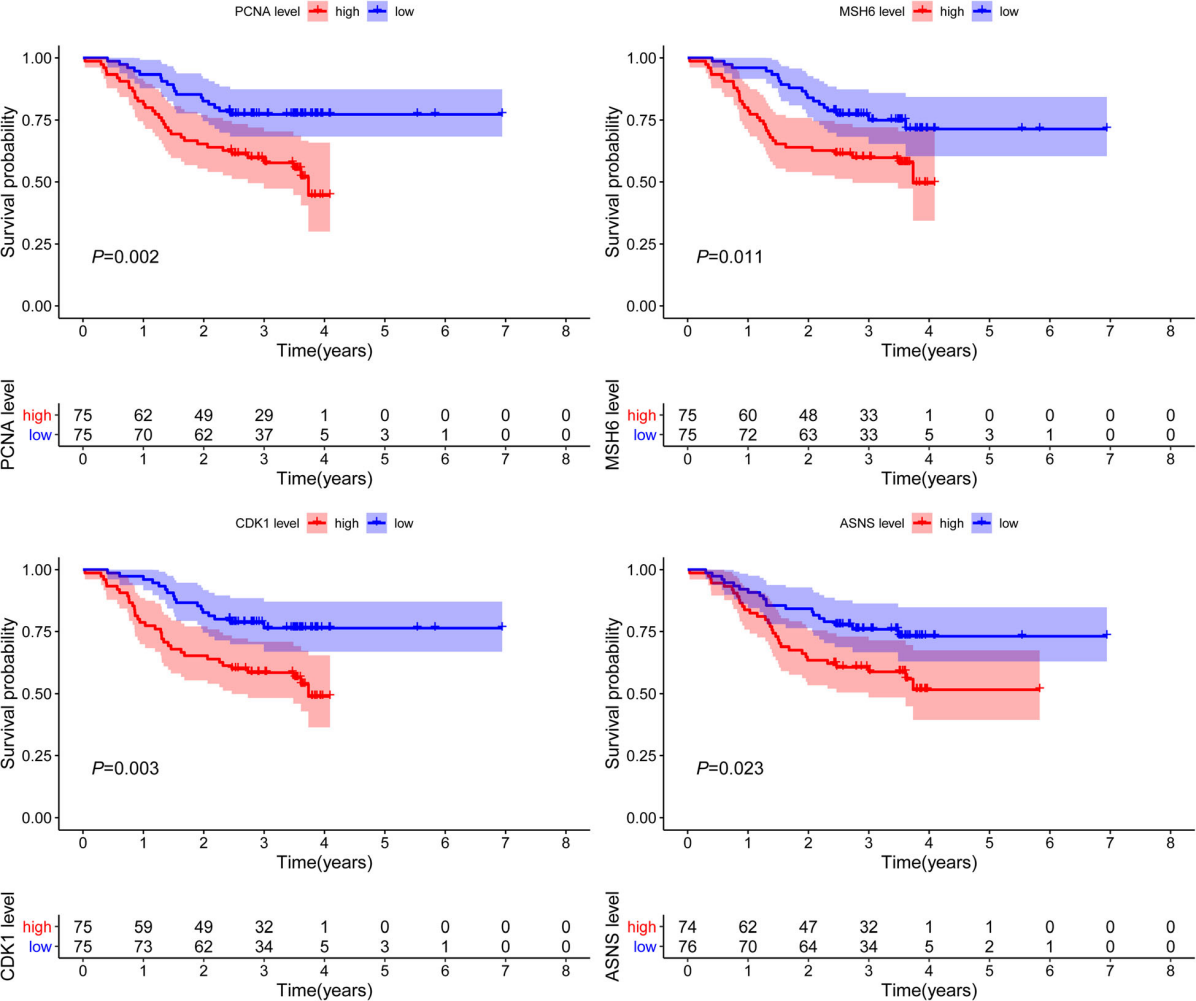
OS analysis demonstrating that the 4 proteins with high had a bad prognosis than that with the low group

Proteomics data was downloaded from TCPA-HCC (level 4; 184 samples and 218 proteins) and combined with clinical data from TCGA. Univariate Cox regression analysis determined the expression of survival-related proteins (Table S10). and we intersect survival-related proteins with CPTAC database, and four survival-related proteins *PCNA, MSH6, CDK1,* and *ASNS* were identified. The Human Protein Atlas (HPA) is a website that involves immunohistochemistry-based expression data for distribution and expression of 20 tumor tissues, 47 cell lines, 48 human normal tissues, and 12 blood cells [15]. In this study, the direct contrast of protein expression of the four genes between normal and HCC tissues was used by immunohistochemistry image and the results are shown in Fig. 5. However, *PCNA, CDK1,* and *ASNS* proteins were not expressed in normal liver tissues but were expressed in high to medium levels in HCC tissues. Besides, *MSH6* was lowly expressed in normal tissues and highly expressed in tumor tissues. TIMER (Differential gene expression module) is a comprehensive asset for systematical investigation of immune infiltrates over various malignancy types. It was used to explore *PCNA, MSH6, CDK1,* and *ASNS* based on thousands of variations in copy numbers or gene expressions in patients with HCC. Similar to our findings, the four proteins were significantly overexpressed in HCC patients in the TIMER database (Fig. 6). OS analysis demonstrated that the four proteins with high had a poorer prognosis than that with a low group (*P* < 0.05) (Fig. 7).

## Discussion

Proteomic analysis of early-stage cancers provides new insights into changes that occur in the early stages of tumorigenesis and represents a new resource for biomarkers for early-stage disease. Proteome characteristics of tumor cells distinguish them from normal cells and are critical in the study of their growth and survival. Proteomic analysis in signaling pathways has become ideal targets for personalized therapeutic intervention in cancer patients [16]. In this study, we identified novel and effective prognostic signatures for patients with HCC. These signatures show great potential in the prognosis prediction of HCC.

In this study, we did a comprehensive analysis of proteomics through CPTAC as well as downloaded proteomic data from TCPA (level 4) which combined with clinical data from TCGA. We first identified 422 differentially proteins and analyzed the function of the identified differentially proteins and then the PPI network construction, we found the most contiguous nodes was *CDK1.* BP was significantly enriched in acid biosynthetic process and catabolic process, MF were mainly enriched in biological compounds binding, CC was mainly enriched in organelles and enzymes and retinol metabolism, chemical carcinogenesis, drug metabolism-cytochrome P450, fatty acid degradation, arginine biosynthesis, PPAR signaling pathway, and other metabolism pathways. A recent study found that Simvastatin can inhibit the HIF-1α/PPAR-γ/PKM2 axis resulting in decreased proliferation and increased apoptosis in HCC cells [17]. Similarly, Wang et al [18] confirmed that the anticancer efficacy of avicularin in HCC was dependent on the regulation of PPAR-γ activities. Therefore, we hypothesis that the differentially expressed proteins identified may play a critical role in drug chemical carcinogenesis via the PPAR signaling pathway, however, there is a need for further studies to confirm this hypothesis. The analysis was restricted to the intersection between CPTAC and TCPA database survival-related proteins and four survival-related proteins *PCNA, MSH6, CDK1,* and *ASNS* were identified.

Proliferating cell nuclear antigen (*PCNA*, also known as *ATLD2*), is a cofactor of DNA polymerase delta which is ubiquitinated in response to DNA damage. A recent study found that *PCNA* knockdown-HepG2 cells under hypoxia showed the induction of more epithelial-mesenchymal transition (EMT) process compared to the control [19]. *PCNA* and EMT-related markers were down-regulated following treatment with Wnt/β-catenin signaling inhibitor (XAV939) and the proliferative activity of HCC cells was significantly inhibited [20]. MutS homolog 6 (*MSH6*) is a member of the DNA mismatch repair MutS family. Togni et al [21] reported a nuclear expression of *MSH6* in HCC excluding a DNA mismatch repair defect and Ozer et al [22] studied the methylation status of *MSH6* involved in DNA repair mechanisms. *MSH6* is associated with an increased risk for breast cancer and should be considered in individuals with a family history of breast cancer [23]. Another study evaluated metachronous colorectal cancer (CRC) incidence according to the *MSH6* gene in Lynch Syndrome (LS) patients who underwent a segmental colectomy [24]. However, there is currently no comprehensive study on the role of *MSH6* in HCC and this study may provide important information for consideration in future studies. Cyclin-dependent kinase 1 (*CDK1,* also known as *CDC2; CDC28A; P34CDC2*), is a member of the Ser/Thr protein kinase family which is essential for G1/S and G2/M phase transitions of the eukaryotic cell cycle. Anti-*CDK1* treatment can boost sorafenib antitumor responses in HCC patient-derived xenograft (PDX) tumor models [25]. Gao et al [26] demonstrated that karyopherin subunit-α 2 (*KPNA2*) may promote tumor cell proliferation by increasing the expression of *CDK1*. Asparagine synthetase (*ASNS,* also known as *TS11; ASNSD*), is involved in the synthesis of asparagine. The expression of *ASNS* has been reported to be high in HCC tumor tissues and closely correlated with the serum AFP level, tumor size, microscopic vascular invasion, tumor encapsulation, TNM stage, and BCLC stage [27]. Li et al [28] found that the expressions of *ASNS* decreased and also functioned as an independent predictor of OS in HCC patients. This study’s OS analysis demonstrated that these four proteins with high had a bad prognosis than those with the low group.

A total of 41 proteins were identified that can serve as an independent prognostic factor for OS. Among the proteins, 8 proteins (*MCM3, MCM7, PCNA, SLC39A1, SMC2, TOP2A, UBE2C,* and *UHRF1*) had AUC value above 0.7. The use of the prognostic model and clinical pathology data can improve the sensitivity and specificity of 1-, 3-, and 5-year OS prediction. The 8 proteins were used to build a prognostic model and final *SLC39A1* and *UBE2C* choose to build the prognostic model. Solute carrier family 39 member 1 (*SLC39A1,* also known as *ZIP1, ZIRTL),* acts as a molecular zipper to bring homologous chromosomes to close apposition [29]. In prostate cancer, zinc levels have been reported to be decreased and the *ZIP1* transporter is lost [30]. Similarly, studies reveal that *hZIP1* (*SLC39A1*) is expressed in the zinc-accumulating human prostate cell lines, LNCaP, and PC-3 [31]. However, the role of *SLC39A1* in HCC remains unknown. Ubiquitin-conjugating enzyme E2 C (*UBE2C*, also known as *UBCH10; dJ447F3.2*) is an enzyme required for the destruction of mitotic cyclins and cell cycle progression. Studies have demonstrated that knockdown of *UBE2C* expression suppresses proliferation, migration, and invasion of HCC cells in vitro. Moreover, the silencing of *UBE2C* also increases the sensitivity of HCC cells to sorafenib [32]. This study was not without limitations. The results have not been validated in clinical samples, and they do not provide accurate clinical data due to the relatively small number of patients used.

## Conclusion

This study established a novel protein signature for HCC prognosis prediction using data retrieved from online databases. However, the signatures need to be verified using independent cohorts and functional experiments.

## Supplementary information

**Additional file 1:Table S1.** The detailed clinical information of CPTAC-HCC patients. Table S2. The 422 differentially expressed proteins identified using the CPTAC database. Table S3. A total of 542 interactions and 236 nodes screened to establish the PPI network. Table S4. The top five most contiguous nodes: CDK1, AOX1, CYP2E1, CYP3A4, and TOP2A. Table S5. Cox regression analysis of the identified 105 survival-related proteins. Table S6. Univariate Cox regression analysis of survival-related proteins. Table S7. Multivariate Cox regression analysis of survival-related proteins and 41 proteins identified as independent prognostic factors for OS. Table S8. ROC curves investigating the use of the protein patterns as early predictors of HCC incidence and the 8 proteins with AUC value above 0.7. Table S9. The relationship between the 8 proteins and clinical factors. Table S10. Univariate Cox regression analysis exploring the expression of survival-related proteins in the TCPA database.

## Data Availability

Data sharing is not applicable to this article as no datasets were generated or analyzed during the current study.

## Abbreviations

HCC: Hepatocellular carcinoma
AFP: Alpha-fetoprotein
CPTAC: Clinical Proteomic Tumor Analysis Consortium
TCPA: The Cancer Proteome Atlas
TCGA: The Cancer Genome Atlas
